# Digital Technology-Based Learning to Facilitate Critical Thinking by Student Nurses in Clinical Practice

**DOI:** 10.3390/nursrep16070226

**Published:** 2026-06-30

**Authors:** Evelyn Lindiwe Ngobeni, Agnes Makhene, Lerato Matshaka

**Affiliations:** 1 Gauteng College of Nursing, Ann Latsky Campus, Auckland Park, Johannesburg 2006, South Africa; lindiwe.mohoaduba@gauteng.gov.za; 2 Gauteng Department of Health Research and Compliance, Johannesburg 2001, South Africa

**Keywords:** digital technology, facilitation, nursing, critical thinking, student nurses

## Abstract

**Background/Objectives**: Critical thinking in healthcare settings is essential in nursing and is a key element of delivering high-quality patient care. However, the disconnect between theoretical knowledge and practical application, along with pressure from workloads, continues to create persistent challenges in nursing education. In this context, the increasing demand for facilitators to cultivate critical thinking skills in students necessitates technological approaches. Digital technologies can supply access to learning materials, clinical case studies, and interactive tasks that reinforce critical thinking principles. They can also support personalized learning paths and provide immediate feedback to learners. **Methods**: The study used qualitative, exploratory, and descriptive methods to achieve its objectives. A purposive sampling technique was used to intentionally select clinical facilitators and student nurses as participants. The sample included eleven female clinical facilitators, two male clinical facilitators, eight female student nurses, and four male student nurses. Data was gathered through semi-structured individual interviews. **Results**: A total of 25 participants were identified and met the eligibility criteria. The study was conducted at a nursing education institution in Gauteng, to get perceptions of both clinical facilitators and student nurses on how critical thinking can be enhanced using digital technology in clinical practice. Study participants perceived that digital technology is a promising strategy to support the development of critical thinking skills. **Conclusions**: The transition to digital technology-based approaches will provide accessibility to various learning platforms across time and location to facilitate the critical thinking and competencies of student nurses.

## 1. Introduction

Critical thinking is an evidence-based, disciplined reasoning that is clear, reasonable, or open-minded (Adhikari, 2025) [1]. Developing critical thinking skills in nursing is essential for building a scientific foundation for the profession and for creating disciplines in which the use of theoretical perspectives is increasingly assessed and applied. According to [2], Critical thinking skills are important for nurses, like other health practitioners, to effectively manage complex care situations in a challenging environment that demands increased accountability. One of the goals of nursing education is to produce nurses with the ability to think critically and thus be able to provide safe nursing care. Upon graduation, nurses must have critical thinking skills in addition to basic nursing and science knowledge to make the appropriate clinical judgment [3]. The development of these skills requires a different teaching and learning strategy. Critical thinking characters and learning styles of student nurses are of primary concern to nurse clinical facilitators because they influence the teaching methods used in their development. Student nurses who think critically in line with creative thinking and innovation will be useful to survive in the dynamics of the 4.0 industrial revolution and beyond [4]. The use of digital technology leads to dynamic work, internationally and in multi-disciplinary environments; therefore, competencies such as flexibility, adaptability, and innovation will facilitate creativity, critical thinking, and change management.

Digital technology has become an indispensable part of student nurses’ daily lives. Students can easily obtain information from the internet without any space or time limitations. In 2022, 81.6% of adults in the US were smartphone users [5]. According to the data by Statistics South Africa and the Independent Communications Authority of South Africa [6], almost fifty-five million persons (96.1%) have a smartphone in South Africa.

Today, we all use digital technology and other information and communication technologies for a variety of social and professional purposes. Digital technology facilitates mobility as well as flexibility for information retrieval [7]. These attributes make it an important approach for clinical nursing education and practice, where access to evidence-based facts is necessary to ensure critical thinking skills and the best possible standards of care. This may be especially factual in the context of community practice, where students are often lacking access to information resources and may be required to make clinical decisions in the absence of facilitator support [8]. With the rapid development of information technologies and mobile devices, digital technology-based learning to facilitate critical thinking has made great progress in nursing education in many countries [9]. 

The utilization of technology differs among various categories of healthcare professionals and in diverse environments, as learning is often more engaging when students can pose questions and debate the findings of experiments documented with smartphones, thereby enhancing their critical thinking abilities [10]. For instance, some studies report that medical journal mobile apps are more commonly used by physicians than by nurses, encouraging active exploration through engaging elements and offering instant access to information that necessitates medical students assessing their significance and reliability, thereby fostering skills in appraisal for critical thinking [5]. Studies have also found that employing digital technology-based applications such as calculators, web-based education, simulations, forums, and virtual learning environments improves nurses’ critical thinking abilities in nursing operations [11]. Many medical mobile apps targeting healthcare professionals to promote evidence-based practice by enabling the evaluation of new information against current knowledge, which can enhance students’ critical thinking and their capacity to interpret, analyze, and draw conclusions from complex data, encouraging a more reflective comprehension instead of simple memorization, are available. Their number is growing [12]. Student nurses could take advantage of the qualities of mobile learning apps with their portability and accessibility to link theoretical knowledge to more complicated real-life situations, which increases their critical thinking, self-confidence and learning efficiency.

Some features of digital technology have become a useful resource for student nurses in the clinical setting [13]; for example, smartphones make it easier to calculate drug doses and access information related to health and clinical tools [14]. For these reasons, digital technology could have significant potential to support student nurses’ critical thinking, as well as improve the care provided to patients [15]. 

However, there is no in-depth information about the quality and credibility of the resources that student nurses use on their mobile devices [16]. In addition, digital technology encourages communication between peers, clinical facilitators, or preceptors, enabling them to share information, experience, or advice, constituting a part of their training. Furthermore, mobile device use can help to improve self-efficacy, critical thinking, clinical competencies, and promote clinical efficiency and subsequently minimize the stress of student nurses during their clinical practice [8]. The latest information needed in clinical work is generally available online but rarely is there a computer or laptop in the clinical work areas. Therefore, this study aimed to explore, using a qualitative approach, the perceptions of clinical facilitators and student nurses of their experiences with the use of digital technology in clinical areas and perceived challenges within clinical nursing education. It was therefore evident that there is a need for a mobile and practical device to access clinical information for the enhancement of the critical thinking of student nurses [17].

## 2. Materials and Methods

### 2.1. Design

This research adopted a qualitative descriptive approach to explore the previously little-examined experiences related to fostering critical thinking through digital technology. This methodological approach was selected because it enables a direct, low-inference summary of occurrences using the participants’ everyday language. Creswell & Creswell describe the qualitative descriptive approach as a comprehensive, flexible, and practical approach designed to provide a straightforward, rich description of events, phenomena or human experiences. It is well-suited to the objective of uncovering practical perceptions for the facilitation of critical thinking in this context [18]. The study was conducted using the thematic analysis process of transcribing, coding, and identifying recurring patterns and insights into how strategies to facilitate critical thinking are perceived by the participants.

### 2.2. Population and Sampling Method

A purposive sampling method was used to select clinical facilitators as the main personnel who facilitate clinical learning for the student nurses. The recruitment followed a two-way pathway: firstly, the nursing education institution under study identified clinical facilitators who accompanied student nurses in their clinical settings during placements for participation in the study and also assisting in recruiting student nurses; secondly, the identified clinical facilitators acted as intermediaries, facilitating access to eligible student nurses in their third year of study and providing details so that those interested in participating voluntarily could contact the researcher. A total of 20 clinical facilitators from the nursing education institution under study, were contacted; of these, 13 agreed to participate in the study and signed the consent forms and 7 declined due to lack of availability. A total of 27 student nurses in the third year of study were identified for participation; of these, 12 agreed to participate and signed the consent forms and 15 declined, citing lack of interest in participating. The established inclusion criteria were clinical facilitators who have 1 or more years of experience in clinical accompaniment of student nurses in clinical areas, and students who are in their third year of study as they have sufficient information on challenges in the clinical areas where they are placed. The sample size was determined based on the sufficiency of the information obtained, which allowed us to adequately address the research question and generate a rich and detailed understanding of how facilitation of critical thinking could be enhanced through digital technology-based learning.

### 2.3. Method of Data Collection

Data collection was conducted by the primary researcher, a female nurse educator holding a master’s degree in nursing education to explore and describe the perceptions of clinical facilitators and student nurses on how critical thinking could be facilitated through digital technology. The researcher has extensive experience in teaching student nurses, which provided relevant contextual understanding of the participants’ environment. Prior to the commencement of the study, participants attended a formal presentation delivered by the researcher, which served to introduce the study’s aims and establish an initial connection. During the semi-structured interviews, the researcher focused on building rapport to ensure a comfortable environment for open dialogue, while remaining mindful of her professional background to mitigate potential bias. The COREQ guidelines were followed to ensure transparency, quality and comprehensive reporting of the semi-structured interviews [19]. Semi-structured individual interviews were used to collect data from the willing participants. This allowed flexibility in the availability and convenience for the participants, and the freedom to express their perceptions in focused areas and settings that were non-threatening [20]. Data was collected during April 2025–July 2025, between Monday and Friday, in the participants’ preferred settings, e.g., the clinic and the facilitators’ offices as per participants’ availability, scheduled appointments, and convenience. The duration of the interviews ranged from forty-five to ninety minutes, and open-ended questions were used to obtain rich data from the participants. No repeat interviews were carried out during this study. The semi-structured individual interviews were conducted to explore and describe the perceptions of the participants by responding to the research data collection question: How can the critical thinking of student nurses be facilitated through digital technologies? Subsequent questions emanated from the participants’ responses for exploration, ensuring the clarity, increased credibility, and authenticity of the data. Field notes and audio recordings were taken for verbatim transcriptions. Data saturation was reached, as the twenty-five participants who volunteered, signed informed consent, and participated in the semi-structured individual interviews provided rich data information.

### 2.4. Data Analysis

The interviews were done through audio recording, and were transcribed verbatim through the Transcribe tool (https://pdfsimpli.com/) for manual transcription of speech-to-text conversion. The matrix-building method of data analysis as guided by [21], a framework which included data consolidation, displaying consolidated data in matrices, drawing, and verifying conclusions, was used. A table was created with columns and rows to arrange the collected data for easy viewing, analysis, and comparison of information. Data collection questions were separated into rows. Information was collected from various participants during interviews, where similar responsive patterns related to the question were clustered and consolidated into a sole source to enrich data on the clustered information. The two groups of participants converge, for a collective, integrated phase, which resulted in a shared understanding or output. Consolidated data were then put together to assist with systematic and extensive data analysis. A constructed matrix framework assisted the researcher with detailed analysis, consolidation, and display, to facilitate the understanding and meaning interpretation of the data. The data was revisited, and initial codes were created from the datasets, which were organized based on meaning and patterns, leading to the identification of themes. The themes were developed and assessed by the author and corresponding authors. The coding process was conducted in an iterative manner, with two supervisors independently coding the data, and any discrepancies were discussed and resolved until a consensus was achieved. Four main themes were developed by verifying and drawing conclusions from the collected and consolidated data.

### 2.5. Ethical Considerations

The study was approved by the ethics committee at the University of Johannesburg, South Africa (REC-3037-2024). All participants were given an information leaflet prior to commencing the study; the participants were well informed about the purpose of the study and signed consent forms to participate in the semi-structured individual interviews; they were also aware of their right to withdraw their informed consent and decline to answer questions asked at any time, without penalty. Continuous reminders of the option to withdraw during interviews were given and their confidentiality and anonymity preservation was ensured. In compliance with the Protection of Personal Information (PoPIA) Act 4 of 2013, all participants’ personal data were anonymized by assigning alphanumeric codes (P1 to P25) before analysis and storage. Data will be stored for five years following publication of the study and subsequently deleted permanently. No AI tool was used to process the participants’ data.

### 2.6. Reflexivity

The trustworthiness of the study was ensured by adhering to the Lincoln and Guba criteria [22]. Transferability was addressed by providing a comprehensive account of the methods and data collection, as well as direct quotations in the presentation of the findings. Conformability was attained through the independent analysis of transcripts by the principal author and corresponding authors, followed by a collaborative session to compare, correlate and discuss emerging themes according to the guidelines established by Holloway and Galvin [23] to ensure the validity and accuracy of the data. The discrepancies related to the study design, data analysis and conclusions were discussed between the researcher and supervisors until a consensus was reached. To reduce interviewer bias, the interviews were carried out by the principal researcher. Concerning reflexivity, the principal researcher has experience as a facilitator in various clinical environments, without any professional ties to clinical teaching; this allowed for a more impartial demeanor during the process of data collection and interpretation. Nonetheless, before starting the study, the researcher and supervisors reflected on their own beliefs and assumptions about facilitating critical thinking, recognizing that these could affect the research process. To counteract this influence, a continuous practice of reflexivity was implemented throughout the study, involving the maintenance of a reflective diary and regular discussion sessions, where emerging interpretations were critically examined and potential biases linked to the researcher’s perspective were explored. The researcher and supervisors concurred with the findings.

## 3. Results

**Figure 1:**
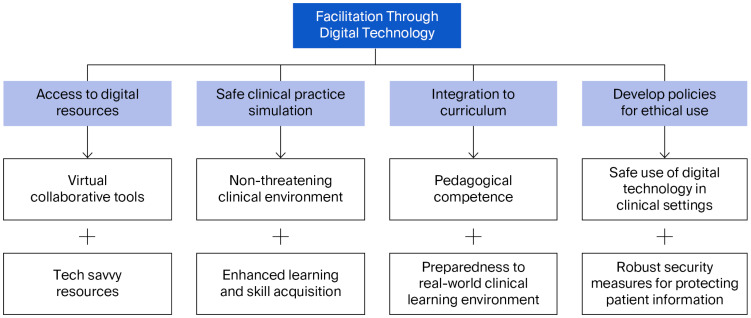
The four thematic areas identified during the analysis, together with their main and secondary categories. Each thematic area comprises two main categories.

Thirteen clinical facilitators and twelve third-year undergraduate student nurses from the nursing education institution under study participated in the study. The clinical facilitators had an average of 9.2 years of clinical facilitation experience. The qualitative analysis of the data was based on the narratives of facilitators (coded with letter P followed by interview number 1–13) and student nurses (coded with the letter P followed by the interview number 14–25). Data was collected until saturation was reached. Table 1 below indicates the demographic data of the participants and Figure 1 presents the four thematic areas discovered during the analysis, along with their primary and secondary categories. Each thematic area consists of two primary categories.

### 3.1. Theme 1: Facilitation Through Access to Digital Resources

Current research emphasizes that simply providing access to technology is insufficient; the way digital tools are integrated into pedagogical practices is what drives CT development. Interactive simulations, personalized learning modules, and collaborative platforms are proven to foster analytical reasoning and problem-solving; however, disparities in access to technology can hinder the equitable development of critical thinking skills [24]. This thematic area consists of two main categories: virtual collaborative tools and tech savvy resources.

#### 3.1.1. Virtual Collaborative Tools

Virtual tools can promote critical thinking by facilitating real-time brainstorming, structured debate, and interactive problem-solving. Participants reported that these tools may enable students to analyze, evaluate, and synthesize information, promoting constructive feedback and deeper, team-based inquiry. Statements from participants demonstrate their perceptions:

- P5: “We often use the Moodle app to test how students engage on online platforms and realized that online discussions with students adds a sense of participation in students’ learning and acquisition of critical thinking skills.”
- P1: “By thinking and reflecting together with students about the questions and connections, can shape their understanding and enhance their critical thinking.”
- P11: “Allowed me to see what others were thinking and allowed me to further develop my understanding of the topic.”
- P9: “It gives students time to process ideas and thoughts more critically.”
- P13: “online discussions are more beneficial for us as students because we must critically think about the topic and create a cohesive response. It requires me to think about the topic, and everything that influences that topic.”
- P4: “I like how the discussions allow them to speak with others in the Moodle class and see how opinions differ among other students. I also like how the discussions let students think about and review topics that we’ve discussed in class.”
- P15: “Online discussion questions place us in different situations and in that way, forces us to combine both the lecture material to real life.”
- P10: “Provision of time to process and reflect; allowing for deeper thinking to understand; and facilitating application and connection-making of course materials.”

These reflections highlight that virtual collaborative tools can support the facilitation of critical thinking for student nurses with technology in the clinical areas. The authors focus on the importance of creating interactive learning environments which can be adaptable to different learning styles and paces. Students considered the use of two forms of technology beneficial in meeting different needs and preferences, offering varied means to actively participate in learning (P13: “*online discussions are more beneficial for us as students because we must critically think about the topic and create a cohesive response. It requires me to think about the topic, and everything that influences that topic*”), and facilitators also believed that the online discussions and reflections would benefit student learning (*P5:* “*we often use the Moodle app to test how students engage on online platforms and realized that online discussions with students adds a sense of participation in students’ learning and acquisition of critical thinking skills.*”)

They valued critical thinking instruction being intentionally aligned with subject-specific content, facilitating understanding, application, and relevance of course material. This adaptability encourages independent thinking and allows students to explore topics in-depth, contributing to the development of critical thinking skills. Interactive learning often incorporates multimedia resources such as videos, simulations, and interactive exercises. They provide diverse stimuli for learners, motivating critical thinking by requiring them to analyze information presented in various formats. Interactive learning can make the learning process more enjoyable and engaging for students. Technologies for working out issues with debate contribute to the development of critical thinking, give an opportunity to determine one’s own position, form the ability to state and defend one’s perspective, deepen knowledge of the discussed problem, and foster the ability to communicate.

Thus, interactive learning provides a dynamic and engaging environment that promotes the development of critical thinking skills by fostering students’ active participation in educational practices. Many participants identified that the inclusion of technology to enhance their learning is critical, as is having a variety of in-class and digital technologies to facilitate their development of critical thinking. The study findings demonstrated that appropriately integrating digital technology into clinical classes to achieve specific objectives has the potential to support the students’ development of critical thinking. Inclusion of such components in clinical learning will help students achieve their learning outcomes.

#### 3.1.2. Tech-Savvy Resources

Technology integration in education has significant potential to develop students’ cognitive skills and shape a generation of critical thinkers. In regions like sub-Saharan Africa, inadequate digital infrastructure directly compromises the development of 21st-century skills. The use of innovative educational tools and applications can stimulate analytical thinking, problem-solving, and creativity. Issues such as disparities in access to technology, varying digital skills among teachers, and the disruption that technology use can cause in the classroom necessitate appropriate strategies and policies. A four-year study conducted in multiple countries (Botswana, Ghana, Kenya) across 15 sites found that while many institutions endorse “learner-centred” digital methods, only a few achieved significant gains in students’ CT. Success was tied to a major shift in facilitator orientation moving from information transmission to active facilitation. Participants reported challenges in clinical areas where limited resources affect their clinical learning. Their experiences are illustrated in the following statements:

- P2: “Technology plays a key role in this process. It can greatly improve our ability to prepare students for future challenges by improving their thinking skills and problem-solving abilities.”
- P8: “By prioritizing the development of critical thinking and creativity, we can make the learning experience more vibrant and empowering for students.”
- P3: “For example, digital platforms that offer up-to-date medical, drug, and disease information for both professionals and patients can be used.”
- P12: “Using innovative teaching methods and technology can help equip students with the skills they need to succeed in a complex world. Digital systems for electronic health records must be integrated with mobile apps for all healthcare professionals’ access.”
- P20: “A lack of Electronic Health Records (EHR) means hours are wasted on manual paperwork, taking time away from patient interaction.”

These narratives show that it is important to explore and implement models of technological integration in education that are effectively designed to support the student nurses’ cognitive skills. Modern education should endeavor not only to improve their digital literacy, but also to equip them with critical thinking skills that will be an important asset in their future professional and personal lives. The facilitators believe that innovative technology must be integrated in clinical areas where students are *placed (P12: “Using innovative teaching methods and technology can help equip students with the skills they need to succeed in a complex world. Digital systems for electronic health records must be integrated with mobile apps for all healthcare professionals’ access”)*, while student also agree that they need electronic health records for efficient documentation of patients’ health outcomes (*P20: “lack of Electronic Health Records (EHR) means hours are wasted on manual paperwork, taking time away from patient interaction”*). Computer programs and technology-based learning applications can improve memory and concentration skills through structured and interactive exercises. In addition, technologies such as Virtual Reality (VR) and Augmented Reality (AR) can be used to create immersive and realistic learning environments, thereby enriching the learning experience and enabling the exploration of complex concepts in a more instinctive and practical way.

### 3.2. Theme 2: Facilitation Through Safe Clinical Practice Simulation

Certain scholars emphasize the significance of promoting critical thinking through safe clinical practice simulations, which establish a standardized patient environment where students can contemplate clinical choices without endangering real patients. Additionally, some of the literature indicates varied results concerning the effect of simulation on critical thinking, underscoring the necessity for more rigorous methods to evaluate its effectiveness. Moreover, there is insufficient research on how simulation effectively bridges the theory–practice divide in developing nations [25].

#### 3.2.1. Non-Threatening Clinical Environment

Participants in both groups frequently noted a clinical environment where students can practice without real-world consequences to be the source that could encourage and promote the facilitation of critical thinking skills. Participants also highlighted that these non-threatening clinical environments could make them feel safe because the use of high-quality mannequins assured them that the outcome would be almost the same as if it were real patients. The following quotations highlight their perceptions:

- P9: “High-fidelity simulation and virtual simulations will provide a safe, nonjudgmental environment for students to practice complex clinical scenarios, recognize patterns, and manage crises.”
- P2: “Structured debriefing after clinical experiences or simulations helps students analyze their actions, understand the ‘why’ behind their decisions, and identify areas for improvement.”
- P6: “I think if the stakeholders (clinical area management) can provide functional equipment and sufficient staffing to prevent high-stress levels, because chaotic situations can become intimidating to our students.”
- P1: “We should encourage a culture where staff can admit uncertainties, ask for help, and voice concerns without fear of bullying or revenge.”
- P19: “I believe that a calm, structured environment reduces the likelihood of medical errors and that we, together with staff, learn faster when we feel safe to explore and ask questions. A respectful, low-stress environment improves patient cooperation and satisfaction.”

#### 3.2.2. Enhanced Learning and Skill Acquisition

Participants described that it is expected of clinical facilitators to display clinical competence in fundamental skills ensuring a safe nursing environment for students to learn and practice new clinical skills before they start working on actual patients within a clinical setting. They also stated that students must be adequately prepared to carry out clinical skills competently and efficiently because clinical competence is obtained through simulated, real-life experiences and repetitive learning opportunities. Their views are quoted below:

- P3: “…clinical facilitators who model critical thinking, provide constructive feedback, and encourage independent thought rather than just delivering content.”
- P7: “Using open-ended, probing questions e.g., “Why,” “What if,” “How do you know?” prompts students to think deeper, analyze information, and justify their clinical decisions.”
- P22: “if we engage in group discussions to solve difficult, unclear clinical problems, I think that can encourage us to work collaboratively and make safe decisions for our patients.”
- P4: “we must encourage a supportive classroom culture where students feel comfortable taking intellectual risks and asking questions without making them feel like we are judging them.”
- P25: “The lecturers should also link theoretical knowledge with practical application to make our learning relevant and improve our critical thinking and problem-solving abilities.”
- P5: “facilitators can engage students in group discussions, debates, and learning communities to expose students to diverse perspectives, building flexibility and improving thinking skills.”
- P11: “our duty is to teach student nurses to question current methods and rely on research findings, ensuring care is safe and up to date.”
- P5: “we can also encourage students to use the nursing process or SWOT analysis (eehhh…. strengths, weaknesses, opportunities, threats) to organize patient data and evaluate management options.”

### 3.3. Theme 3: Integration into Curriculum

Successful integration of digital technology into the curriculum necessitates a transition from a teacher-focused model to one centered around students, incorporating critical thinking at every stage of nursing education by linking theoretical understanding to practical, complex clinical situations [26].

#### 3.3.1. Pedagogical Competence

Integration of theory and practice can be enhanced by clinical facilitators through promoting learning aimed at advancing cognitive, psychomotor, and affective skills. Pedagogical competence requires using diverse, active teaching methods that move beyond traditional, lecture-dominated approaches. During interviews, both groups of participants agreed that facilitation of critical thinking using high-fidelity simulation for safe practice can help students identify what they did well and where they need to improve. Participants’ perceptions are shown in the following statements:

- P12: “…so we have to shift from lecture-based, passive learning to active engagement through discussions, debates, and collaborative teamwork for student nurses.”
- P6: “Ma’am, ehh… I think some facilitators may not have adequate training and competency in teaching or facilitating critical thinking, so continuous professional development and workshops are required to ensure the college can effectively implement these strategies.”
- P23: “Yes, if we have a learning environment where students are genuinely valued and encouraged to be independent, it can create a way for building our critical thinking skills.”
- P10: “I think that the nursing curriculum should reflect the integration of best practices that have been implemented in clinical practice to foster critical thinking, be strategically placed in any course, and incorporated into any learning setting.”
- P14: “……if we can change the clinical setting into an environment that persuades and reinforces critical thinking as crucial in nursing education, we can shift away from the traditional paradigm of presenting course content.”
- P7: “and those lectures, and slide presentations can confine students to a limited perspective of receiving and memorizing information instead of promoting analysis of the concepts; therefore, we need critical thinking exercises that can change the student focus from remembering to active learning.”
- P18: “These digital tools can help us link new information to existing knowledge, assisting in prioritizing care and visualizing difficult patient scenarios.”

These views from facilitators and student nurses emphasize that nursing educators must shift from teacher-dominated methods of teaching to actively learning strategies to foster students’ critical thinking ability. This involves planning and implementation of strategies throughout the nursing curriculum and developing a framework of best practices for promoting critical thinking in nursing education which can assist nursing institutions with integrating critical thinking strategies into nursing curricula.

#### 3.3.2. Preparedness for Real-World Clinical Learning Environment

The nursing curriculum needs to be aligned to the clinical setting to ensure that graduates are equipped to face the challenges of complex and dynamic healthcare delivery systems. Digital technology tools enhance clinical reasoning, decision-making, and judgment, preparing learners for real-world environments through experiential, repeatable scenarios. Participants agreed that the use of digital technologies is important if they are to be competent professionals in the real world.

- P9: “I think that clinical facilitators should model critical thinking, provide constructive feedback, and encourage critical thinking rather than just delivering content to students.”
- P3: “we can let students observe expert clinician procedures remotely, providing insights into real-world environments to promote their critical thinking skills.”
- P19: “another thing is to develop physical examination training by overlaying digital information, helping in the understanding of anatomy and clinical procedures using telehealth tools.”
- P17: “Digital tools allow students to apply theoretical knowledge to complex lifelike scenarios before entering the actual clinical setting, so yes we need them in clinical settings.”
- P2: “Interactive technologies can facilitate the analysis of data, helping students interpret patient information, make informed decisions, and adapt to changing conditions.”

Nursing education institutions have a crucial role in ensuring digital tools are used to improve the clinical learning of student nurses (*P17: “Digital tools allow students to apply theoretical knowledge to complex lifelike scenarios before entering the actual clinical setting”; P2: “Interactive technologies can facilitate the analysis of data, helping students interpret patient information, make informed decisions”*). While technology is valuable, it should complement, not replace, in-person clinical experience. Over-reliance on digital tools should be monitored to not reduce the capacity for deep, independent, critical analysis, making it important to maintain a focus on critical thinking skills.

### 3.4. Theme 4: Development of Policies for Ethical Use

The development of policies for the ethical use of digital technologies to facilitate critical thinking in clinical practice requires a framework that balances technological innovation with patient safety, data privacy, and the preservation of human judgment [27]. As digital tools become deeply integrated into healthcare and nursing education, policies must guide users toward actively evaluating AI-generated data rather than passively accepting it, fostering critical thinking skills essential for modern nursing and clinical decision-making.

#### 3.4.1. Safe Use of Digital Technology in Clinical Settings

The secure application of digital technology in healthcare environments consists of combining resources such as electronic health records (EHRs) and artificial intelligence with strong cybersecurity measures, comprehensive staff training, and well-defined governance to minimize human errors and improve patient care. Essential practices involve safeguarding data privacy, confirming the precision of technology, having backup systems ready for service interruptions, and promoting an ongoing culture of safety monitoring. Participants explained that it is crucial to have safely useable digital technology in the following statements:

- P11: “We need protocols for safeguarding electronic medical records and patient data, particularly when using mobile or AI-integrated devices.”
- P3: “The NEIs, together with clinical area management, must establish and agree on guidelines that will guide users not to override established clinical standards and treatments against AI-recommended treatments.”
- P24: “……these policies should ensure that AI acts as an assistant, with final decisions resting with qualified professional nurses and doctors to uphold accountability.”
- P16: “I think we need clear guidelines for the use of social media and messaging systems to maintain patient confidentiality and professional boundaries.”
- P15: “there is a need for policies to be co-created by clinical managers, clinicians, facilitators, students, patients, and IT experts as part of professionals working with patient information to ensure comprehensive, real-world relevance.”

Continuous monitoring and auditing must be implemented using algorithm vigilance tools to regularly audit these tools or systems for bias, errors, and performance implications; this was highlighted by participants *(P11: “We need protocols for safeguarding electronic medical records and patient data, particularly when using mobile or AI-integrated devices.”; P24: “these policies should ensure that AI acts as an assistant, with final decisions resting with qualified professional nurses and doctors to uphold accountability.”)*. Development of protocols to inform patients when AI or digital tools are being used to analyze their data or assist in their care, and providing options to opt out, should be included when making these policies.

#### 3.4.2. Robust Security Measures for Protecting Patient Information

Security for protecting patient information during clinical critical thinking involves implementing secure, up-to-date software/hardware, firewalls, and data anonymization, to ensure confidentiality and integrity while supporting safe, evidence-based care decisions. Participants describe this as per the statements below:

- P20: “Keep antivirus software updated, and use secure, private, and auditable network connections.”
- P4: “When these electronic health records are available, I think that users can have access to health records to make timely decisions, but this must be protected by strict access logs and monitoring.”
- P14: “For the security of our patients’ information, we must ensure the integrity of data through regular audits and validation, crucial for accurate clinical reasoning and critical thinking of our students.”
- P3: “We can also discuss amongst ourselves that we secure the messaging and compatible systems for communication among team members to avoid using unsecured channels.”
- P11: “… and as lecturers, we should maintain and clearly communicate to our students the safety measures in place, allowing them to understand how their data informs their patient care.

The participants suggest that continuous improvement in privacy practices among healthcare providers, through ongoing training and comprehensive privacy frameworks, is vital for enhancing patient confidentiality and supporting effective care coordination (*P20: “Keep antivirus software updated, and use secure, private, and auditable network connections”; P4: “we must ensure the integrity of data through regular audits and validation”).*

## 4. Discussion

This qualitative study finding indicated that the perceptions of clinical facilitators and student nurses on facilitation of critical thinking included needing digital resources to enhance the students’ critical thinking when placed in clinical facilities. The students described the challenges of a lack of resources that can enable them to acquire information to make clinical decisions for their patients. While clinical facilitators agree with the lack of resources being a challenge, they described how it will be of importance to guard against improper use of technology if there are no monitoring systems on the correct use of the digital tools. Digital technology-based approaches to facilitate critical thinking for student nurses in a nursing education institution in Gauteng must be developed. The findings were outlined as promoting the use of digital resources for their access to information and promoting safe clinical practice through simulation and integrating digital technology in the curriculum and instruction. The transition to digital technology-based approaches will provide access to various learning platforms across time and location to facilitate the critical thinking and competencies of student nurses. Nursing education institutions will be guided by the approaches in planning, designing, and delivering interactive digital technology-supported learning activities that promote student-centered learning and develop their competencies in preparation for clinical practice.

Digital technology usage plays a vital role in the facilitation of learning in higher education institutions [28]. It is the backbone of teaching and learning as information searching, teaching, learning, and assessment are all reliant on digital technology [29]. Students use digital technology and digital technology devices for web browsing and class preparation and to document and record class proceedings for referrals. Students reported that digital technology will allow them to have access to a range of websites that enables them to critically think when making on-the-spot clinical decisions at clinical facilities for work-integrated learning (WIL). Furthermore, integration of digital technology usage with clinical settings has proven to be the driver of ideas in improving critical thinking skills [30].

With the constantly changing landscape of healthcare, clinical facilitators are faced with many challenges in preparing students for the increasing complexities of the work environment. The current expanded use of computers and information systems in healthcare means that all healthcare workers, especially nurses, will need to interface with multiple technological sources to either enter or extract data to aid them in caring for patients [31]. This highly technological environment demands that student nurses are taught to think critically and exercise clinical decision-making while delivering safe quality care. This sentiment is echoed by clinical facilitators and it is noted that professional nurses need critical thinking skills, independent clinical judgment, management and organizational skills, leadership abilities, and technological understanding to practice in the varied healthcare settings that exist [32].

It is therefore important to educate student nurses on how to use available technologies to access evidence-based data that supports their critical thinking as well as on how to input and retrieve data pertinent to patient care [33]. The national nursing and midwifery education strategy aimed to embed informatics in pre-registration curricula to prepare nurses to deliver digital technology-enabled care. It also emphasized that post-registration education and continuing professional development should ensure nurses can embrace digital technology and innovation. This means both pre- and post-registration nurses need to have adequate digital knowledge and skills to enable them to be competent practitioners not only in the National Health Service (NHS) in South Africa but also if they go to work abroad.

From the analysis of data and conceptualization of the findings within the relevant literature, digital technology-based approaches were developed to facilitate critical thinking for student nurses in a nursing education institution in Gauteng. The analysis of the participants’ perceptions assisted in the development of digital technology-based approaches to facilitate critical thinking for student nurses. The description of the methodology and demographics of the participants will assist prospective researchers in evaluating the transferability of the study and findings to another context.

### Limitations

The study was conducted in a nursing education institution in Gauteng that provides education and training for the undergraduate nursing programs at a diploma level; therefore, the findings cannot be generalized.

## 5. Conclusions

The study’s findings confer a need to maximize the impact of digital technology on critical thinking. Nursing institutions should integrate interactive, high-fidelity simulations that are accompanied by structured debriefing sessions within the curriculum. The study also adds to the body of knowledge. It is relevant as it deals with the benefits of using digital technology to facilitate critical thinking for student nurses in clinical practice.

The study highlights the critical role of hybrid learning methodologies, which promote self-directed and flexible learning styles, complemented by facilitator support and continuous engagement in the clinical setting. Additionally, the significance of integration of technology in the clinical context was evidenced by the participants’ perceptions, as it represents a real-world environment in which students can encounter and recognize the complexities involved in their practical application within healthcare. The findings support the incorporation of digital technology as a mandatory strategy within clinical education, thereby enhancing critical thinking and information literacy skills, and ensuring alignment between the nursing education institution and clinical settings.

## Figures and Tables

**Table 1:** Demographics of the participants.

ID	Gender	Age
Facilitators		
P1	Female	54
P2	Female	33
P3	Female	35
P4	Male	47
P5	Female	39
P6	Female	58
P7	Female	36
P8	Female	44
P9	Female	43
P10	Female	51
P11	Male	47
P12	Female	41
P13	Female	43
Students		
P14	Male	24
P15	Female	22
P16	Female	27
P17	Female	23
P18	Male	24
P19	Male	24
P20	Female	22
P21	Female	27
P22	Female	23
P23	Male	24
P24	Female	24
P25	Female	22

## Data Availability

The data presented in this study are available on request from the corresponding author due to privacy and ethical restrictions.

## Public Involvement Statement

No public involvement in any aspect of this research.

## Guidelines and Standards Statement

This manuscript was drafted against the Consolidated Criteria for Reporting Qualitative Research (COREQ) [19].
